# Time-course profiling of behavioral phenotypes and Ywhah/ERK signaling expression within the vestibular region of the brainstem during motion sickness habituation

**DOI:** 10.3389/fnins.2026.1955920

**Published:** 2026-10-07

**Authors:** Xue Yue, Chenxi Jia, Lihong Zhai, Jie Deng, Dongjun Yang, Zhanguo Jin

**Affiliations:** 1 Anhui Medical University, Hefei, Anhui, China; 2 Air Force Medical Center, PLA, Beijing, China

**Keywords:** brainstem, motion sickness habituation, neurophysiology, neuroplasticity, proteomics

## Abstract

Motion sickness habituation represents a core neuroplastic model for investigating vestibular disorder adaptation, yet its brain-region-specific temporal molecular mechanisms remain poorly defined. Here, we adopted multi-brain-region proteomic screening combined with time-series molecular validation to characterize key molecular dynamics during motion sickness habituation. Adult mice received 15 consecutive days of sinusoidal rotational stimulation, and model establishment was verified via four behavioral indices. A self-constructed five-dimensional weighted scoring system based on proteomic data confirmed that the vestibular region of the brainstem exhibited the most prominent molecular response among the hippocampus, cerebral cortex, and brainstem vestibular region. Candidate molecules were further screened by integrating network centrality, statistical significance, functional relevance, druggability, and neurological disease correlation. Reverse transcription quantitative real-time PCR and Western blot were performed to detect the expression changes of candidate proteins in brainstem vestibular region tissues of the complete time-series restraint-only control group and habituation group. Temporal validation revealed progressive downregulation of Ywhah and a time-dependent increase in the ratio of phosphorylated Erk1/2 to total Erk1/2 in the vestibular region of the brainstem. The opposite expression trends of Ywhah and p-Erk1/2/total Erk1/2 first reached statistical significance on Day 5, indicating temporal coupling. In summary, this study delineates distinct temporal molecular patterns in the brainstem vestibular region during motion sickness habituation, with overlapping critical time windows for Ywhah suppression and ERK activation. These findings provide reliable longitudinal molecular evidence and promising candidate targets for further exploring the neuroplastic mechanisms of vestibular habituation.

## Introduction

In vestibular disorders, motion sickness (MS) induced by physical movement is predominantly explained by the sensory conflict hypothesis. This theory proposes that mismatched neural circuits arise when peripheral integrated sensory signals conflict with internal predictive signals generated by the brain. Activated sensory conflict inputs can be suppressed via inhibitory neuronal circuits to weaken aberrant afferent signaling. Alternatively, repetitive identical motion stimulation enables recalibration of internal predictive signals to align with peripheral sensory integration, defined as motion sickness habituation (Reason and Brand, 1975). Notably, researchers hypothesized sensory conflict-selective vestibular neurons responding preferentially to passive motion as early as 1982, yet direct experimental evidence remained absent for decades (Oman, 1982). Recent work by Oman and Cullen (2014) characterized this neuronal subtype within the vestibular nuclei contained in the primate brainstem and deep cerebellar nuclei. During active head movement, afferent signals from semicircular canals and otolith organs are eliminated via an internal comparator, which contrasts predicted self-motion against peripheral sensory feedback, residual uncompensated signals encode passive motion information.

Multiple palliative interventions are available for vestibular motion sickness, motion sickness habituation driven by neural plasticity carries Grade A clinical evidence and represents the most widely adopted effective strategy (Koch et al., 2018). Motion sickness habituation is defined as the gradual attenuation of autonomic motion sickness responses after repeated identical vestibular stimulation. This adaptive process differs fundamentally from vestibular rehabilitation training targeting central vestibular compensation in both conceptual definition and underlying mechanisms, and this study mainly focuses on motion sickness habituation.

Substantial evidence has demonstrated that the hippocampus, brainstem, and cerebral cortex are key central regions regulating autonomic responses, spatial orientation and motion perception in motion sickness, and they participate in generating and updating internal models of self-motion and spatial positioning (Balaban, 1996; Bergman et al., 1994; Uno et al., 2000). During learning and adaptive modulation, neurotransmitter-dependent plasticity elevates the firing frequency of specific brainstem neurons, reshaping brainstem-mediated reinforcement learning and enabling stimulus-specific responses to distinct motion inputs. As such, the brainstem, where vestibular nuclei reside, serves as a critical hub for vestibular signal integration and plasticity linked to habituation (Arber and Costa, 2022), exploring protein expression within the vestibular region of the brainstem provides critical insights into sensorimotor integration and vestibular processing. Motion sickness habituation induced by repeated motion stimulation represents an ideal model for investigating central neuroplasticity. Systematically characterizing the temporal dynamics of molecular events across brain tissues throughout habituation constitutes an essential prerequisite for deciphering the neural plasticity mechanisms underlying motion sickness habituation.

Within brain tissue, activation of the vestibular region is modulated by multiple neurotransmitters including acetylcholine, glutamate, histamine, and γ-aminobutyric acid (Zanni et al., 1995). Studies have demonstrated that activated molecular interactions can alter intracellular calcium ion (Ca^2+^) levels, thereby triggering activation of mitogen-activated protein kinase (MAPK) family proteins (Chan et al., 2005; Kupzig et al., 2005), including the cholinergic system (Joseph et al., 2007; Kanno et al., 2003). The 14–3-3η protein encoded by the Ywhah gene belongs to the highly conserved 14–3-3 protein family. Highly abundant in brain tissue, this protein mediates signal transduction by binding to serine/threonine phosphorylation residues and participates in multiple biological processes including apoptosis (Buret et al., 2016; Chen et al., 2025), tumorigenesis (Li et al., 2024; Liu B. et al., 2023; Wang et al., 2024), and metabolic homeostasis, rendering it a promising molecular candidate for neurological disorder research (Chen et al., 2025; Wang et al., 2024). Furthermore, Ywhah interacts with MAPK pathway proteins to co-modify messenger RNA (mRNA) and protein abundance. This interaction inhibits osteogenic differentiation of bone marrow mesenchymal stem cells and modulates pathogenic mediators linked to rheumatoid arthritis, while also participating in cellular remodeling and inflammatory responses (Liu Y. Q. et al., 2023; Maksymowych et al., 2014). Nevertheless, the specific functional contribution of Ywhah within the vestibular region of the brainstem during motion sickness habituation remains unclear, and the reciprocal regulatory interaction between brain neurotransmitters and Ywhah remains poorly characterized.

Collectively, the above evidence led us to hypothesize that the brainstem acts as the prioritized brain region governing motion sickness habituation, and that Ywhah and MAPK pathway-associated molecules exhibit detectable temporal expression shifts over the course of habituation. In the present study, quantitative proteomic profiling was performed to identify the brain region with the most prominent molecular response to habituation. We further applied Gene Ontology (GO) and Kyoto Encyclopedia of Genes and Genomes (KEGG) analyses to annotate candidate molecules and their corresponding signaling pathways in the vestibular region of the brainstem, and validated the temporal expression patterns of these candidate molecules using brain tissues harvested from motion sickness model mice.

## Materials and methods

### Animals

This experimental protocol was approved by the Ethics Committee of the Air Force Medical Center, PLA, Beijing (2025-04-PJ01). Six-week-old male specific pathogen-free (SPF) C57BL/6J mice, obtained from Beijing Vital River Laboratory Animal Technology Co. Ltd., were used as experimental subjects. The animals were housed in a controlled environment with a 12-h light/dark cycle, appropriate temperature and humidity, and free access to food and water. All procedures were conducted in accordance with the National Institutes of Health Guide for the Care and Use of Laboratory Animals. Animals were randomly divided into a rotation habituation group and a restraint-only control group. The rotation habituation group (*n* = 8 per group) included the control group (Control), habituation Day 1 group (Day 1), habituation Day 5 group (Day 5), habituation Day 10 group (Day 10), and habituation Day 15 group (Day 15); the restraint-only control group included Sham-Day 1, Sham-Day 5, Sham-Day 10, and Sham-Day 15. The Control and Sham-Day 1 groups underwent identical procedures. From each group, three mice were randomly selected for brain tissue collection to complete the experiments.

All animals underwent three consecutive days of acclimation training prior to formal experimentation, including adaptation to the vestibular apparatus fixation, open-field environment, and temperature measurement procedures. Both acclimation and formal rotation sessions were conducted daily at 17:00 under constant temperature and humidity.

### Rotation device and procedures

Using a mouse vestibular-ocular reflex testing instrument (GAT-MVOR939, Gianttek Technology, China), motion sickness habituation training was completed on eight mice (Figure 1a). Conscious mice were secured in the matching restraint apparatus, with the rotation frequency set at 0.8 Hz, angular velocity at 40 °/s, and sinusoidal oscillation for 5 min per session over 15 consecutive days. This device enables synchronous recording of the slow-phase velocity of nystagmus during motion stimulation (Figure 1b). Immediately after each rotation session, the number of fecal pellets and urination status were recorded, and tremor behavior was observed (Figure 1b). Since the restraint apparatus caused mechanical indentations on the fur, piloerection could not be reliably quantified and was therefore excluded from subsequent statistical analysis. Mice were then transferred to a sterile white open-field arena measuring 50 cm × 50 cm × 50 cm, and the total distance traveled during 5 min of free activity was recorded using an automated tracking system (EthoVision XT 17.5, Noldus, the Netherlands). Body temperature was measured using an animal-specific thermometer, with two repeated measurements taken per mouse and the average value used for statistical analysis (Rahman and Luebke, 2024; Wang et al., 2012; Wang et al., 2017; Wei et al., 2011; Zhang et al., 2016).

**Figure 1:**
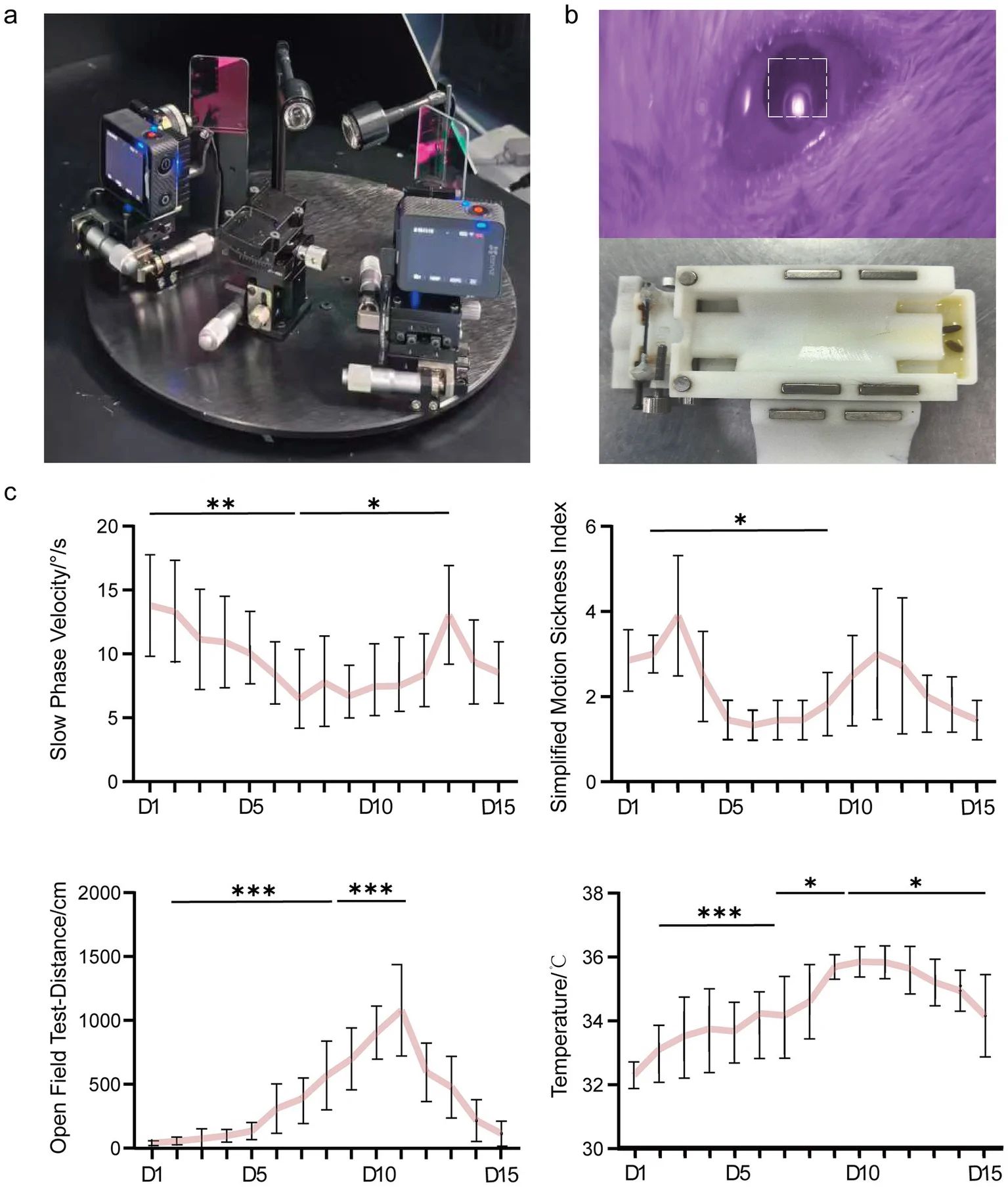
Temporal changes in four behavioral indicators during motion sickness habituation. **(a)** Schematic diagram of the mouse vestibular eye movement instrument; **(b)** Representative images of nystagmus recording and post-rotation defecation and urination behaviors; **(c)** Temporal dynamics of four behavioral indicators throughout the habituation process. All data are presented as the mean ± SD with *n* = 8. Repeated-measures one-way ANOVA was used to evaluate the overall effect of training time on each indicator. Tukey HSD *post hoc* tests were performed to compare daily behavioral data with the Day 1 baseline. *p < 0.05, **p < 0.01, ***p < 0.001 versus Day 1.

The above four indicators were recorded after daily training, with the first training day data set as baseline, and longitudinal analysis of behavioral changes across training was performed using a repeated-measures design. All operators received standardized training to ensure proficient equipment operation and strict compliance with unified behavioral assessment protocols.

### Brain tissue preparation

Following the completion of habituation training at each designated time point, mice were sacrificed for tissue collection. For molecular biology validation experiments and proteomic analysis, mice were euthanized via rapid decapitation. Referring to the fourth edition of the Paxinos and Franklin mouse brain stereotaxic atlas, using a 1 mm mouse brain slicing matrix, and guided by anatomical landmarks including the fourth ventricle, inferior cerebellar peduncle, and medial longitudinal fasciculus, brainstem tissue blocks containing bilateral vestibular nuclei were dissected at the pontomedullary junction, approximately Bregma −5.40 mm to −7.10 mm. Dissected tissues were immediately snap-frozen in liquid nitrogen and preserved at −80 °C. Among these, samples for quantitative proteomic analysis were obtained from the control group and the Day 15 group, designated as “pre” and “aft,” respectively. Tissues included the vestibular region of the brainstem, hippocampus, and cerebral cortex.

### Protein extraction

The sample was taken from −80 °C. An appropriate amount of the sample was weighed and placed in a pre-cooled mortar. Liquid nitrogen was added, and the sample was thoroughly ground into powder. The lysis solution was added and ultrasonic lysis. The homogenate was centrifuged at 12,000 × *g* for 5 min at 4 °C; the centrifugation time could be extended to 10 min when necessary. The supernatant was subsequently collected, and the protein concentration was quantified using a bicinchoninic acid (BCA) assay kit (Solabio). Protein lysates were then adjusted to an appropriate concentration, mixed with 5 × loading buffer, and denatured by incubation in a heating block at 100 °C for 10 min.

### Trypsin digestion

Equal amounts of protein lysates were supplemented with lysis buffer to a consistent volume. Proteins were precipitated by the addition of 1-fold volume of pre-cold acetone, followed by thorough vortexing and supplementation with additional 4-fold cold acetone. After incubation at −20 °C for 2 h, the mixture was centrifuged at 4500 rpm for 5 min. The supernatant was discarded, and the pellet was washed with pre-cold acetone and air-dried naturally. The dried pellet was resuspended in 200 mM tetraethylammonium bromide (TEAB) solution and processed with ultrasonication. Subsequently, proteins were digested overnight with trypsin (50-fold volume). For reduction and alkylation, dithiothreitol (DTT) was added for incubation at 56 °C for 30 min, followed by the addition of iodoacetamide (IAA) to a final concentration of 11 mM. The mixture was incubated at room temperature in the dark for 15 min to complete alkylation.

### Liquid chromatography-mass spectrometry analysis

Peptide samples were dissolved in 0.1% formic acid aqueous solution and separated using an ultra-high-performance liquid chromatography system with linear gradient elution: 4%–7.5% mobile phase B (0.5–0.6 min), 7.5%–30% B (0.6–8.1 min), 30%–44% B (8.1–9.6 min), 44%–55% B (9.6–10.0 min), 55%–99% B (10.0–10.5 min), and 99% B (10.5–12.0 min) at a flow rate of 600 nL/min. Eluted peptides were ionized via a capillary ion source at 1.6 kV and then analyzed using a timsTOF HT mass spectrometer. Data acquisition was performed in dia-PASEF (data-independent parallel accumulation serial fragmentation) mode. A total of 12 PASEF scans were acquired per full MS spectrum covering an m/z range of 300–1,500. Secondary MS analysis was conducted within 15 discrete windows across an m/z range of 395–1,080.

### GO and KEGG enrichment and protein–protein interaction network analysis

After data acquisition, the data were collected using DIA-NN (v1.8) software, searching against the *Mus musculus* reference proteome from UniProtKB/Swiss-Prot (release 2024_12, containing 17,236 sequences). By adding a decoy database and setting a precursor FDR of 1% to control the peptide identification error rate, protein identification and quantitative analysis were performed, yielding the relative quantitative values of all proteins in different samples. Protein information was extracted based on the eggNOG database (v5.0.2).^1^ Pathway enrichment analysis of the expressed proteins was conducted using KAAS^2^ and KEGG Mapper (v5.2).^3^ Protein identification was performed via BLAST alignment (blastp, e-value ≤ 1e-4). Differentially interacting proteins and their relationships were obtained by comparison with the STRING protein–protein interaction network database (v12.0).^4^ On the basis of Fisher’s exact test, Benjamini-Hochberg FDR correction was applied to control the false discovery rate for multiple comparisons. Functional terms with Fold enrichment > 1.5 and *p* < 0.05 were considered significant (Zhu et al., 2024).

### Establishing an objective and efficient screening model

The ratio of the mean R values of proteins in different groups was defined as fold change (FC), and the *p*-value as response significance. Principal component analysis (PCA) was conducted on the R values of the three studied brain regions to identify the differential proteins and specific proteins in each brain region. STRING database, GO and KEGG annotations were utilized to quantify a brain region screening model across five evaluation indicators including PCA separation, Differential Proteins, Unique Proteins, Protein Interactions and Functional Score. We assigned 15% weight to each of the three dimensions of PCA separation, Differential Proteins, and Unique Proteins, and 27.5% weight to Protein Interactions and Functional Score. All continuous variables were normalized before weighted calculation to eliminate dimensional interference.

Subsequently, within the protein–protein interaction network of differentially expressed proteins from the prioritized brain region, interacting proteins were identified using a confidence score threshold of > 0.7 (high confidence). Target proteins were quantitatively screened based on five criteria: Network Centrality, Response Significance, presence in enriched pathways, Drug Target potential (DrugBank Online, DrugBank, https://go.drugbank.com/), and Disease Association (Online Mendelian Inheritance in Man, OMIM, https://www.omim.org/).

### RNA extraction

A suitable quantity of tissue samples was taken out of storage at −80 °C. For homogenization, 1 mL of TRIzol reagent and an appropriate number of grinding beads were then added. Following room temperature incubation, 200 μL of chloroform was added, and the mixture was centrifuged for 15 min at 12,000 rpm and 4 °C. After gathering the aqueous phase, it was combined with an equivalent amount of isopropanol. The mixture was centrifuged for 10 min at 4 °C and 12,000 rpm. After discarding the supernatant, the RNA was periodically cleaned with pure ethanol and centrifuged for 5 min at 12,000 rpm and 4 °C. The RNA was allowed to air dry at room temperature after the supernatant was disposed of.

### Real-time quantitative reverse transcription PCR(RT-qPCR)

Total RNA was reverse-transcribed into complementary DNA (cDNA) using the Evo M-MLV Reverse Transcription Kit (Accurate Biology, China) following the manufacturer’s instructions. RT- qPCR assays were performed with the SYBR Green Pro Taq HS Premix qPCR Kit (Accurate Biology, China) according to the kit protocol. The primer sequences (Biomed, China) used are as follows: Mvb12b: F: 5′-ATGGGCAGAGTGCCAAGAAA-3′, R: 5′-TTAGGGAGATGTGCCTGGGA-3′; Csnk1e: F: 5′-AAGCTCGAATGTGTGAAGACG-3′, R: 5′-TGACCATCACGTTATAGTCTCCC-3′; Uqcr11: F: 5′-GGACTGGGTGCCTTACATCAACG-3′, R: 5′-GTGTCCAGCTTCCTCAGTGTCTTC-3′; Ywhah: F: 5′-TGGAAACTGACACAGCGACA-3′, R: 5′-GGCTGCTGAAAGCTCACAAG-3′; Chmp6: F: 5′-CAAGAAGCAGAGCCGAGTCA-3′, R: 5′-TGCTGCTTCAGTTGCAGGAT-3′; Srsf9: F: 5′-AGGACATGGAATATGCTCTGC-3′, R: 5′-GATGTAGGAAGTCTCACCCTCG-3′; Vps37a: F: 5′-GGAACTCCCATTCCAGTATAGC-3′, R: 5′-TCCAAGATCTGAGTGCATTGT-3′; Utp15: F: 5′-CGGCTCACTGGATAGGAAAGT-3′, R: 5′-AGCTGTGGACGACTTTGTAGG-3′; β-actin: F: 5′-GGCTGTATTCCCCTCCATCG-3′, R: 5′-CCAGTTGGTAACAATGCCATGT-3′ (Bjorkman et al., 2019; Ko et al., 2024; Lin et al., 2023; Sekar et al., 2022; Xiao et al., 2025). The relative mRNA levels of target genes in each sample were calculated using the ΔΔCt method.

### Western blot

Proteins were transported to a polyvinylidene fluoride (PVDF) membrane at a continuous current of 300 mA after being separated by electrophoresis at 120 V. After blocking the membrane for 2 hours at room temperature with 5% skim milk, there were two TBST washes. The primary antibody was added to the membrane and gently shaken throughout the entire night at 4 °C.

Rabbit anti-Ywhah (15222-1-AP, Proteintech, 1:5,000), rabbit anti-Erk1/2 (4695T, Cell Signaling Technology, 1:1,000), rabbit anti-p-Erk1/2 (4370T, Cell Signaling Technology, 1:2,000), and rabbit anti-Alpha Tubulin (80762-1-RR, Proteintech, 1:50,000). Goat Anti-Rabbit IgG (HY-P83646, MedChemExpress, 1:5,000) was the secondary antibody utilized.

### Statistical analysis

All data were presented as the mean ± standard deviation (SD). Statistical analyses were performed using GraphPad Prism 9.5 (GraphPad Software, USA). For all experiments, one-way ANOVA with Tukey HSD *post hoc* analysis was used for multi-group comparisons, and independent-sample t-test was used for two-group comparisons. Behavioral longitudinal data were analyzed using one-way ANOVA to assess overall time effects, with Tukey HSD *post hoc* test comparing daily data to Day 1 baseline. For proteomic analysis, Benjamini-Hochberg FDR correction was applied to control the false discovery rate for multiple comparisons. Statistical significance was defined as *p < 0.05, **p < 0.01, and ***p < 0.001.

## Results

### Establishment of the motion sickness habituation model

To verify the successful construction of motion sickness habituation model, four behavioral indicators were continuously monitored throughout the 15-day training procedure. The simplified motion sickness index gradually decreased from 3.0 ± 0.4 on Day 2 to 1.4 ± 0.4 on Day 8 (Figure 1c). Repeated-measures one-way ANOVA revealed that training time significantly affected the simplified motion sickness index. The statistical result was *F*(14, 105) = 6.210 with *p* < 0.001. Significant differences were identified starting from Day 8 when compared with Day 2, and all differences reached the statistical threshold of *p* < 0.05. The slow phase velocity gradually decreased from 13.7 ± 3.9 °/s on Day 1 to 7.4 ± 3.8 °/s on Day 11 (Figure 1c). Repeated-measures one-way ANOVA confirmed a significant effect of training time on this behavioral index. The corresponding statistical value was *F*(14, 105) = 3.640 with *p* < 0.001. The index decreased significantly from Day 7 relative to the Day 1 baseline, with all differences at *p* < 0.01. The index was significantly higher on Day 13 than on Day 7 (*p* < 0.05). The open field test distance was 37.0 ± 29.3 cm on Day 1 and recovered to 1086.0 ± 361.3 cm in the late stage of training (Figure 1c). Repeated-measures one-way ANOVA verified significant modulation by training time. The statistical outcome was *F*(15, 112) = 54.44 with *p* < 0.001. The open field test distance increased significantly from Day 8 onward relative to Day 1 (*p* < 0.001) and was significantly higher on Day 11 than on Day 8 (*p* < 0.001). Body temperature gradually stabilized from 32.3 °C ± 0.4 °C on Day 1 to 35.8 °C ± 0.4 °C on Day 9 (Figure 1c). Repeated-measures one-way ANOVA also indicated a significant main effect of training time. The calculated value was *F*(15, 112) = 16.73 with *p* < 0.001. Body temperature was significantly higher on Day 6 than on Day 1 (*p* < 0.001), significantly higher on Day 9 than on Day 6 (*p* < 0.05), and significantly lower on Day 15 than on Day 9 (*p* < 0.05). Collectively, the progressive improvement of the four behavioral indicators across the 15-day training period demonstrated the successful establishment of the motion sickness habituation model.

For the slow phase velocity of nystagmus, the first significant alteration relative to the Day 1 baseline appeared on Day 7, and further significant changes were detected on Day 13 relative to Day 7. For the open field test distance, significant elevation first occurred on Day 8 compared with Day 1, and additional significant differences emerged on Day 11 compared with Day 8. The behavioral inflection points of the two indicators fully covered Day 5 and Day 10. Based on these behavioral characteristics, Day 1, Day 5, Day 10 and Day 15 were selected as key time points for subsequent molecular validation. This time-point selection was based on behavioral phenotypic changes and covered the critical stages of initiation, development and maintenance during motion sickness habituation.

### Multivariate quantitative analysis revealed the prioritized regulation of motion sickness habituation resides in the vestibular region of the brainstem

Based on the mass spectrometry data files after acquisition, the database search quality control results showed that the peptide lengths of hippocampal, vestibular region of the brainstem, and cerebral cortex samples were distributed between 7 and 20 amino acids (Figure 2a), and most proteins were identified by two or more peptides (Figure 2b). The first two principal components, PC1 and PC2, in the hippocampus explained 45.2% of the total variance, according to PCA of all data. The 95% confidence interval ellipses for groups were fully separated (Figure 2c). In the vestibular region of the brainstem, PCA showed no overlap between the two group ellipses, and PC1 and PC2 explained 45.8% of the variance (Figure 2d). The 95% confidence ellipses entirely coincided in the cerebral cortex, where the total explained variance was 41.6% (Figure 2e). Proteins with an intergroup R value yielding a t-test adjusted *p*-value < 0.05 were defined as differentially expressed proteins. We defined proteins with *p* < 0.05 and FC > 1.5 as significantly upregulated proteins, and those with FC < 1/1.5 as significantly downregulated proteins, then counted the number of differentially expressed proteins in each brain region. The total numbers of differentially expressed proteins in the hippocampus, vestibular region of the brainstem, and cerebral cortex were 17, 129, and 46, respectively (Figure 2f). The Venn diagram displayed one shared differentially expressed protein between the hippocampus and vestibular region of the brainstem, and six shared differentially expressed proteins between the vestibular region of the brainstem and cerebral cortex. The vestibular region of the brainstem contained 122 unique differentially expressed proteins, the largest number among the three brain regions (Figure 2g).

**Figure 2:**
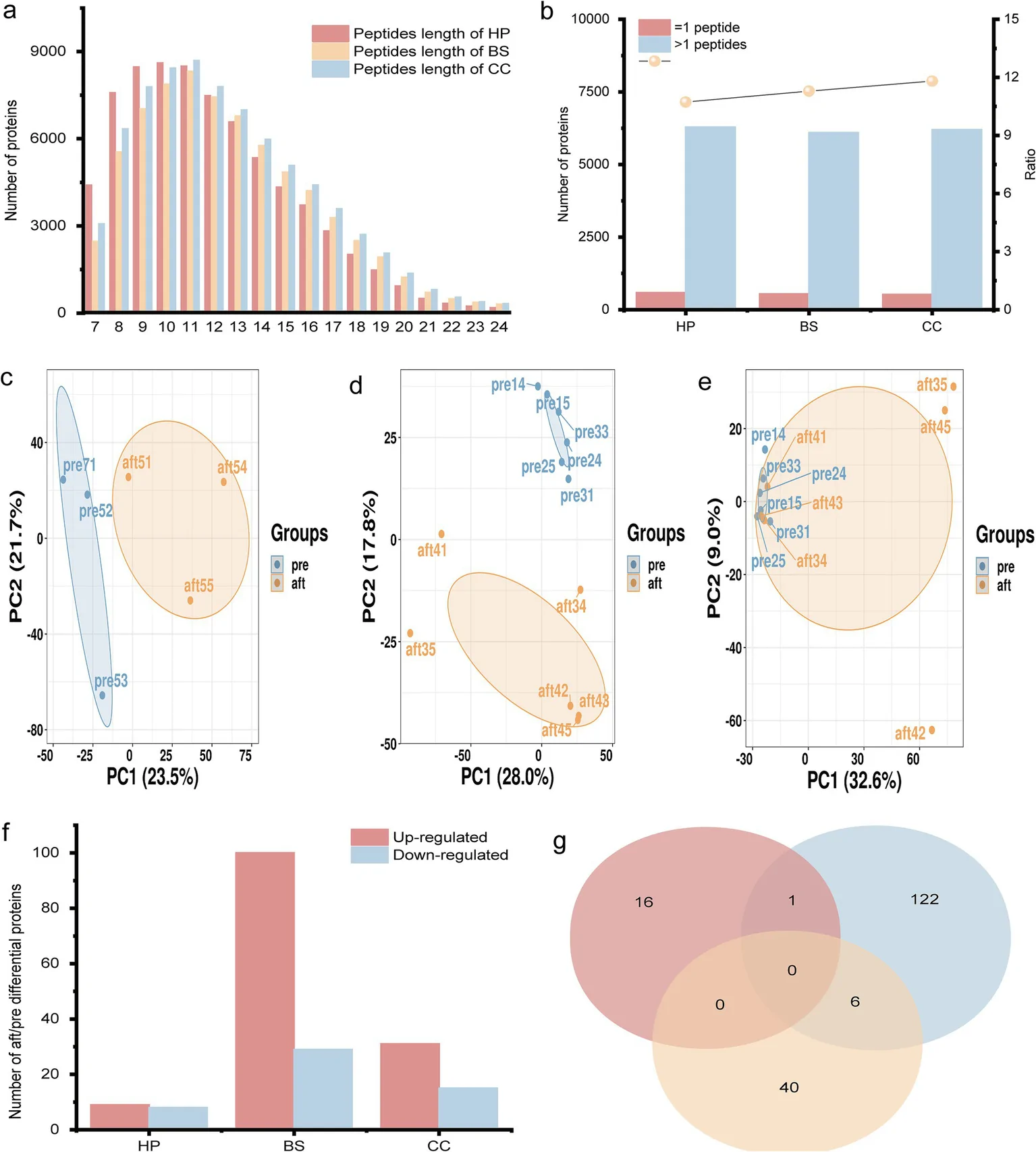
Quantitative protein maps of the hippocampus (HP), vestibular region of the brainstem (BS), and cerebral cortex (CC). **(a)** Peptide length distribution in the three brain regions; **(b)** Proportion of proteins identified by more than one peptide in the three brain regions; **(c–e)** PCA results of the hippocampus, vestibular region of the brainstem, and cerebral cortex; **(f)** Bar chart of upregulated and downregulated differentially expressed proteins in the three brain regions; **(g)** Venn diagram of differentially expressed proteins across the three brain regions, with the hippocampus at upper left, vestibular region of the brainstem at upper right, and cerebral cortex at the bottom.

The interaction networks for the differential proteins in each brain region were retrieved via alignment with the STRING protein–protein interaction database (confidence score > 0.7). A total of 31 interacting proteins were identified in the vestibular region of the brainstem network (Figure 3a), and 6 in the cerebral cortex (Figure 3b), while differential proteins in the hippocampus failed to form a detectable interaction network (Figure 3c). KEGG classification analysis of all differentially expressed proteins from the three brain regions revealed the protein numbers assigned to secondary functional categories under six primary classifications, including Cellular Processes, Environmental Information Processing, Genetic Information Processing, Human Diseases, Metabolism, and Organismal Systems (Figures 3d–f). Protein functional terms including Nervous system, Sensory system, Environmental adaptation, Signal transduction, Endocrine system, Immune system, Development and regeneration, Cell growth and death, and Signaling molecules and interaction exhibited high relevance to motion sickness habituation (Cohen et al., 2006; Hua et al., 2024; Naito, 2003; Wang et al., 2012; Zaback et al., 2025). Quantitative scoring was performed for the three brain regions based on functional relevance and the number of corresponding differentially expressed proteins. The total scores of secondary functional categories were 27 for the hippocampus, 213 for the vestibular region of the brainstem, and 61 for the cerebral cortex (Figure 3g).

**Figure 3:**
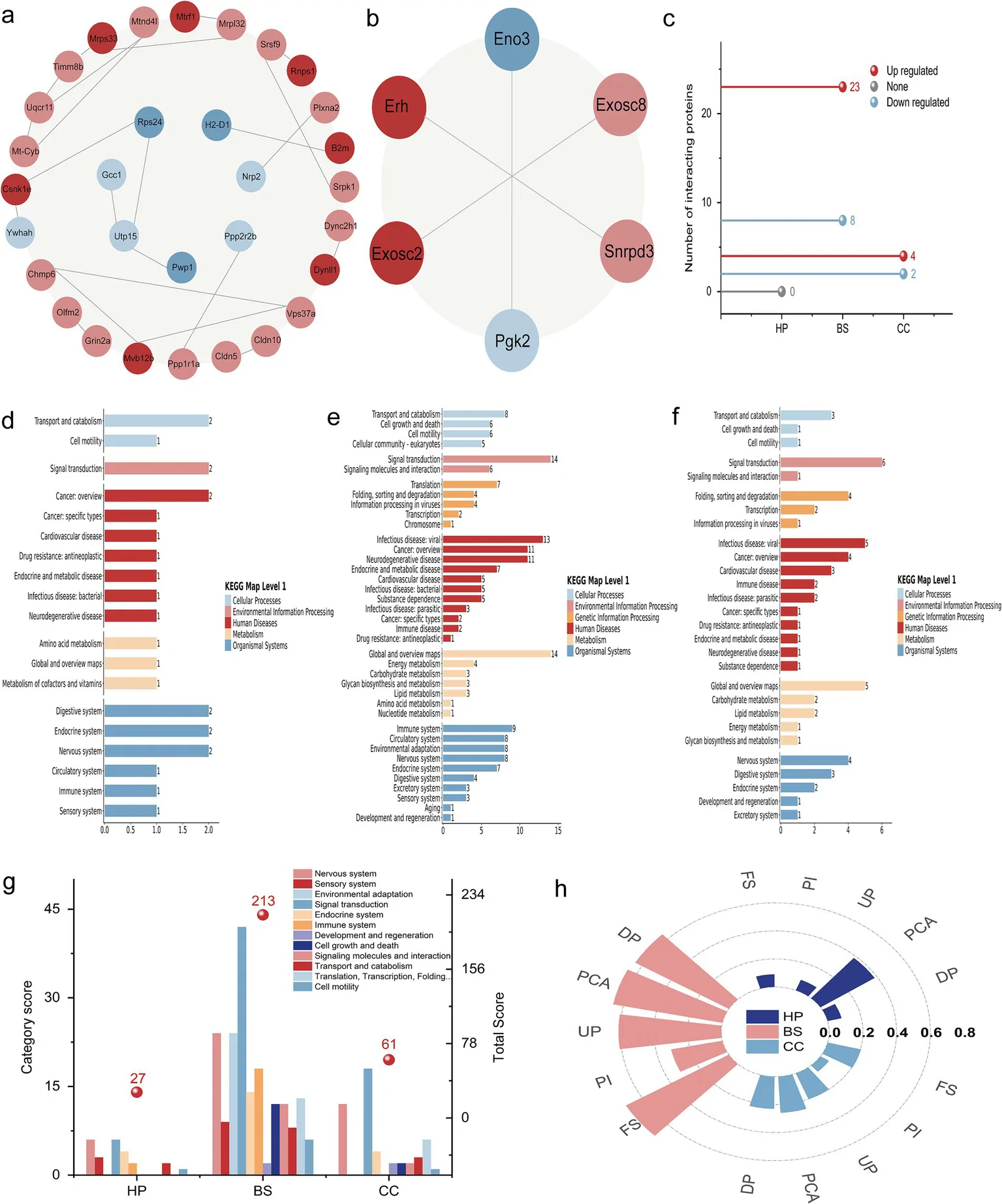
Quantification of protein levels in the hippocampus (HP), vestibular region of the brainstem (BS), and cerebral cortex (CC) and brain region screening model. **(a)** Vestibular region of the brainstem protein interaction network diagram; **(b)** Cerebral cortex protein interaction network diagram; **(c)** Scatter plot of protein interaction counts across three brain regions; **(d–f)** KEGG functional classification bar charts for differentially expressed proteins across three brain regions; **(g)** Functional score bar charts for proteins across three brain regions. The score of each brain region was calculated as the product of the functional weight ratio of motion sickness habituation-related proteins and the number of corresponding proteins. According to the legend order, the weight value was set to 3 for the first four functions, 2 for the subsequent five functions, and 1 for the remaining functions; **(h)** Bar chart of the screening model for the three brain regions established based on principal component analysis (PCA), differential proteins (DP), unique proteins (UP), protein–protein interactions (PI), and Functional Score (FS).

For each brain region, weights of 0.15, 0.15, 0.15, 0.275, and 0.275 were assigned to the five indicators including PCA separation, Differential Proteins, Unique Proteins, Protein Interactions, and Functional Score, respectively. After weighted calculation and normalization, the comprehensive scores were 0.107 for the hippocampus, 0.568 for the vestibular region of the brainstem, and 0.176 for the cerebral cortex. Comparative analysis indicated that the vestibular region of the brainstem was the optimal brain region for screening candidate proteins related to motion sickness habituation (Figure 3h).

### Enrichment and standardized identification of candidate genes for habituation

Five evaluation criteria including Network Centrality, Response Significance, Functional Relevance, Drug Target, and Disease Association were adopted to quantitatively screen and validate the 31 differentially interacting proteins derived from the vestibular region of the brainstem. The network centrality of each interacting protein within the vestibular region of the brainstem was reflected by the number of connecting edges (Figure 3a). Mdm1, Mal2, Grcc10, Tmem87b, and Dtd2 were the top five significantly upregulated proteins in the vestibular region of the brainstem, whereas Avp, Tgfbi, H2-D1, Wdr33, and Bak1 were the top five downregulated proteins (Figure 4a). Proteins including Mal2, Ly6e, Fxyd7, Scrn3, and Nudt4 ranked highly in terms of adjusted *p*-value statistical significance (Figure 4b). One point was assigned for adjusted *p*-value < 0.05, and two points for adjusted *p*-value < 0.001. GO Cellular Component terms were mainly enriched in the MHC protein complex, MHC class I protein complex and semaphorin receptor complex. GO Molecular Function terms included adenyl deoxyribonucleotide binding, dATP binding and acetylcholine receptor inhibitor activity. Biological Process terms were predominantly enriched in positive regulation of dipeptide transmembrane transport, positive regulation of dipeptide transport and regulation of dipeptide transport (Figure 4c). Significantly enriched KEGG pathways contained mmu05031 Amphetamine addiction, mmu05160 Hepatitis C, mmu05030 Cocaine addiction and mmu04962 Vasopressin-regulated water reabsorption (Figure 4d). Each differentially interacting protein was scored as 1 if it existed in significantly enriched pathways and 0 if not. Eighteen interacting proteins including Ywhah, Srsf9, Utp15, and Mvb12b obtained a score of 1. Alignment against the DrugBank and OMIM databases revealed that Ywhah and Nrp2 were associated with both drug targets and human neurological diseases. Thus, the candidate proteins underlying motion sickness habituation in the vestibular region of the brainstem, such as Ywhah, Nrp2, Uqcr11, Csnk1e, Srsf9 and other candidate proteins (Figure 4e), were identified via the multi-criteria scoring approach (Figure 4f).

**Figure 4:**
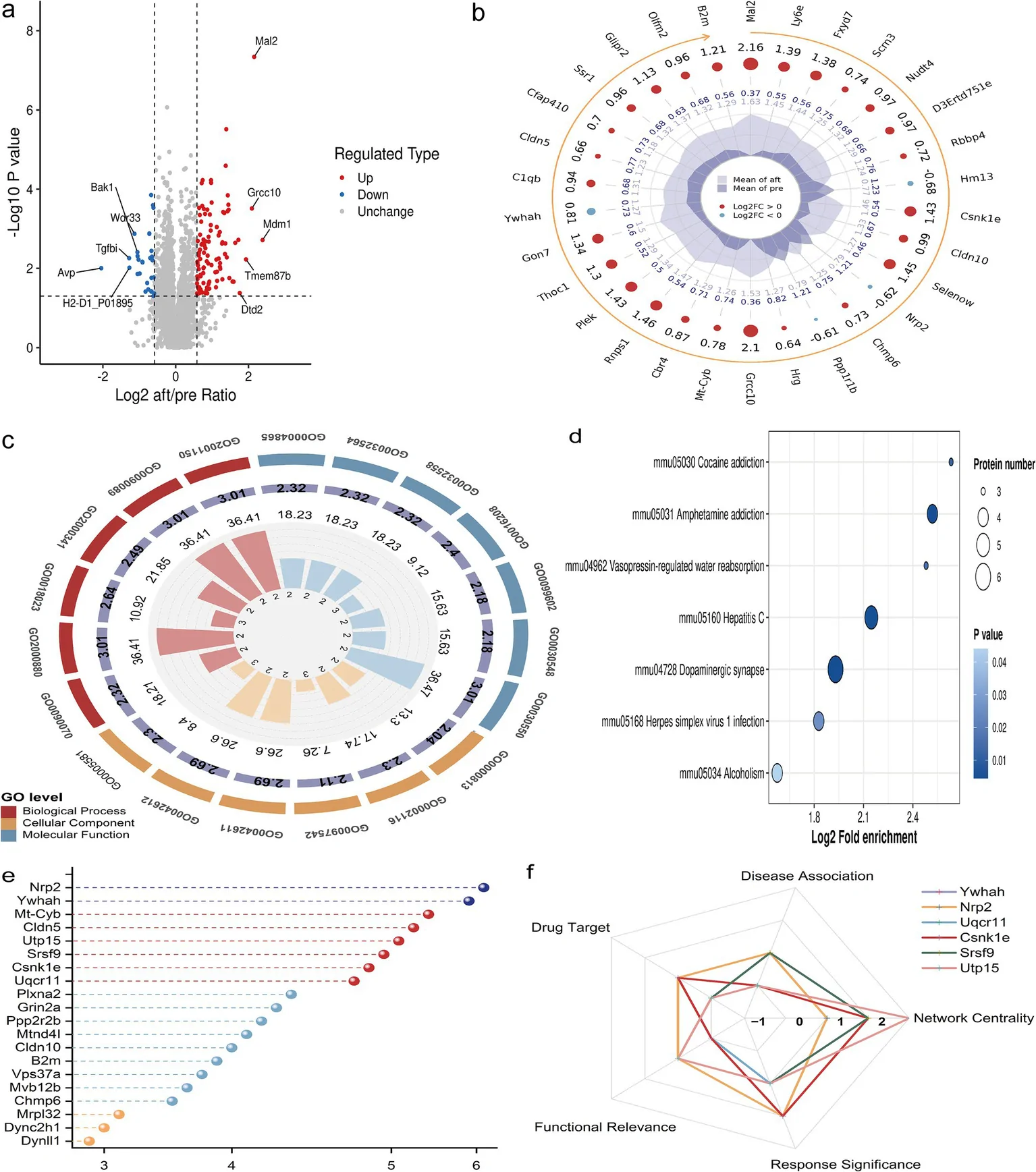
Candidate protein screening model of the vestibular region of the brainstem. **(a)** Volcano plot of differentially expressed proteins in the vestibular region of the brainstem. Horizontal axis: Log_2_-transformed fold change; vertical axis: −Log_10_-transformed adjusted *p*-values. Red, upregulation; blue, downregulation; gray, no significant difference; **(b)** Radar chart of differentially expressed proteins in the vestibular region of the brainstem. Adjusted *p*-values are sorted clockwise in ascending order. The second ring shows Log_2_-transformed fold change (red for upregulation, blue for downregulation; larger dots represent higher fold change), and the third ring shows the mean quantitative levels of the two groups; **(c)** Circos plot for GO enrichment in the vestibular region of the brainstem. Tracks from outside to inside: enriched GO terms, −Log_10_-transformed adjusted *p*-values, enrichment level bar chart, and the count of differentially expressed proteins per term; **(d)** KEGG enrichment bubble plot of the vestibular region of the brainstem. Vertical axis: KEGG pathways; horizontal axis: Log_2_-transformed enrichment level; dot size corresponds to the number of differentially expressed proteins in each pathway; **(e)** Scatter plot of screening scores for candidate proteins in the vestibular region of the brainstem; **(f)** Radar chart of individual scores for the top five candidate proteins in the vestibular region of the brainstem.

### Validation in vestibular region of the brainstem tissue shows decreased Ywhah expression

RT-qPCR was performed to preliminarily verify the mRNA expression of candidate proteins in the vestibular region of the brainstem. One-way ANOVA revealed significant overall differences in mRNA expression across experimental time points with *F* = 5.069 and corresponding degrees of freedom set at 15 and 32. Compared with the control group, the Day 15 group exhibited significantly decreased mRNA levels of *Ywhah*, *Srsf9*, and *Csnk1e*, whereas *Utp15* expression was significantly upregulated. No significant differences were observed for other candidate genes (Figure 5a). Among these, the transcriptional level of *Ywhah* was consistent with the proteomic results and simultaneously satisfied multiple screening criteria, including proteomic effect size, network centrality, pathway relevance, association with neurological disease, and drug target information. Therefore, Ywhah protein was selected as the most suitable candidate protein. Western blot results demonstrated that Ywhah protein expression gradually decreased throughout the motion sickness habituation time series. One-way ANOVA confirmed the significant main effect of training time on Ywhah protein abundance with *F* = 9.925 across degrees of freedom 4 and 10. Significant downregulation was first detected on Day 5 compared with the control group (*p* = 0.0196), and expression remained significantly lower on Day 10 and Day 15 relative to Day 1 (*p* = 0.0494, *p* = 0.0048) (Figures 5b,d). To verify whether this downregulation is specific to motion sickness habituation, Ywhah protein expression was further examined in the restraint-only control group. The results showed no significant differences in Ywhah protein expression among time points in the restraint-only control group (Figures 5c,e). Collectively, Ywhah expression was continuously downregulated from Day 5 to Day 15 during motion sickness habituation.

**Figure 5:**
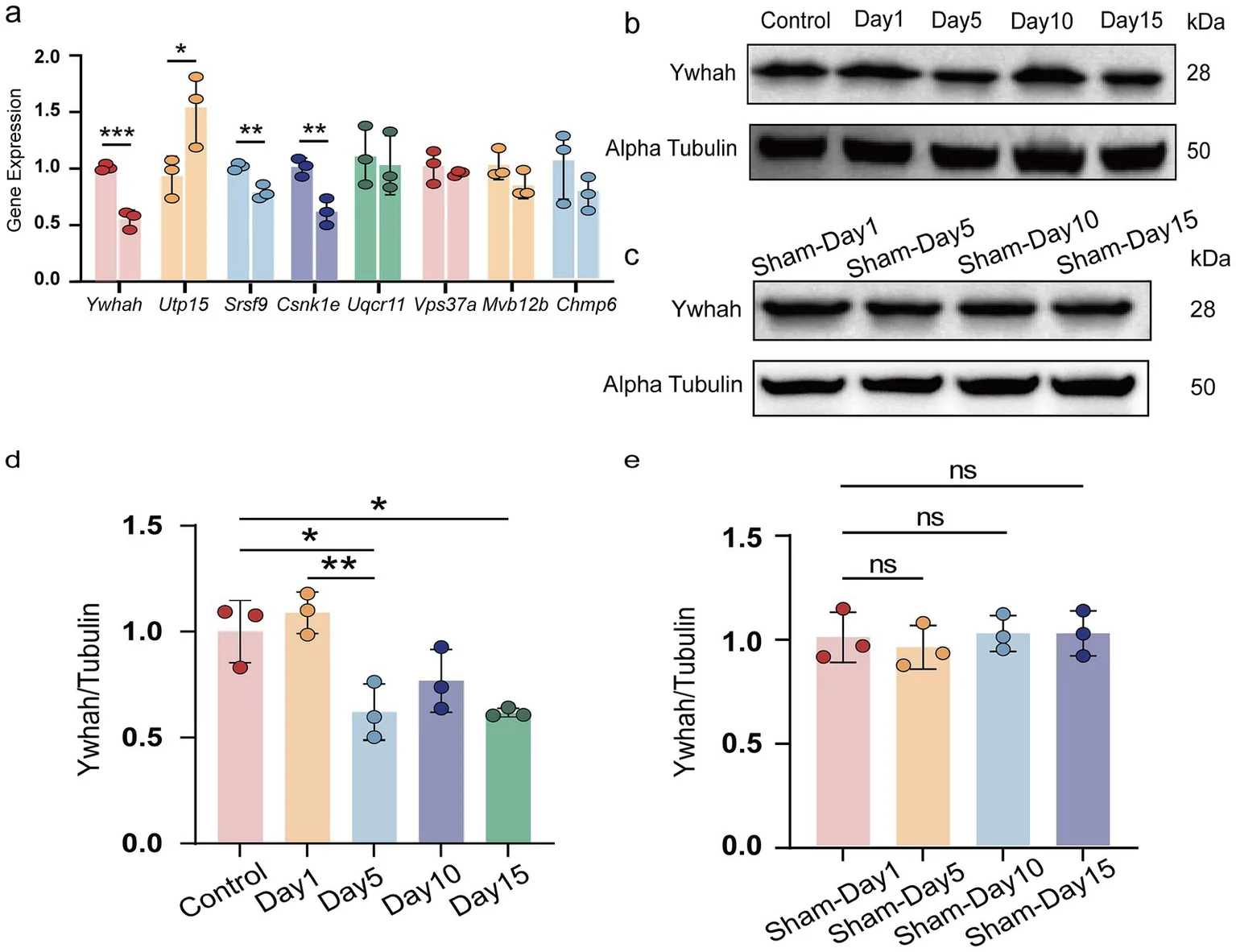
Effects of motion sickness habituation on protein expression in the vestibular region of the brainstem. **(a)** mRNA expression levels of candidate genes in the vestibular region of the brainstem; **(b)** Western blot images of the rotation habituation group; **(c)** Western blot images of the restraint-only control group; **(d)** Changes in Ywhah protein expression across the habituation time series; **(e)** Changes in Ywhah protein expression across the time series in the restraint-only control group. All data are presented as mean ± SD (*n* = 3). Statistical analyses were performed via one-way ANOVA followed by Tukey HSD *post hoc* test. *p < 0.05, **p < 0.01, ***p < 0.001.

### Erk1/2 phosphorylation ratio exhibits an opposite temporal trend to Ywhah

Hierarchical clustering was performed on relevant functional terms based on *p*-values from enrichment pathway analysis (Figure 6a). Significant differences were observed in the mmu05160 Hepatitis C, mmu04728 Dopaminergic synapse and mmu05031 Amphetamine addiction pathways (*p* < 0.01). The validated candidate protein Ywhah was enriched in the mmu05160 Hepatitis C pathway and regulates the MAPK signaling cascade (Figure 6b). Western blot results (Figure 6c) revealed that the ratio of phosphorylated Erk1/2 to total Erk1/2 gradually increased across the habituation time series. One-way ANOVA confirmed significant temporal variation in this phosphorylation ratio with *F* = 15.79 and degrees of freedom of 4 and 10. This ratio increased significantly at Day 5 relative to the control group (*p* = 0.0002) and remained significantly elevated at Day 15 (*p* = 0.008) (Figure 6d).

**Figure 6:**
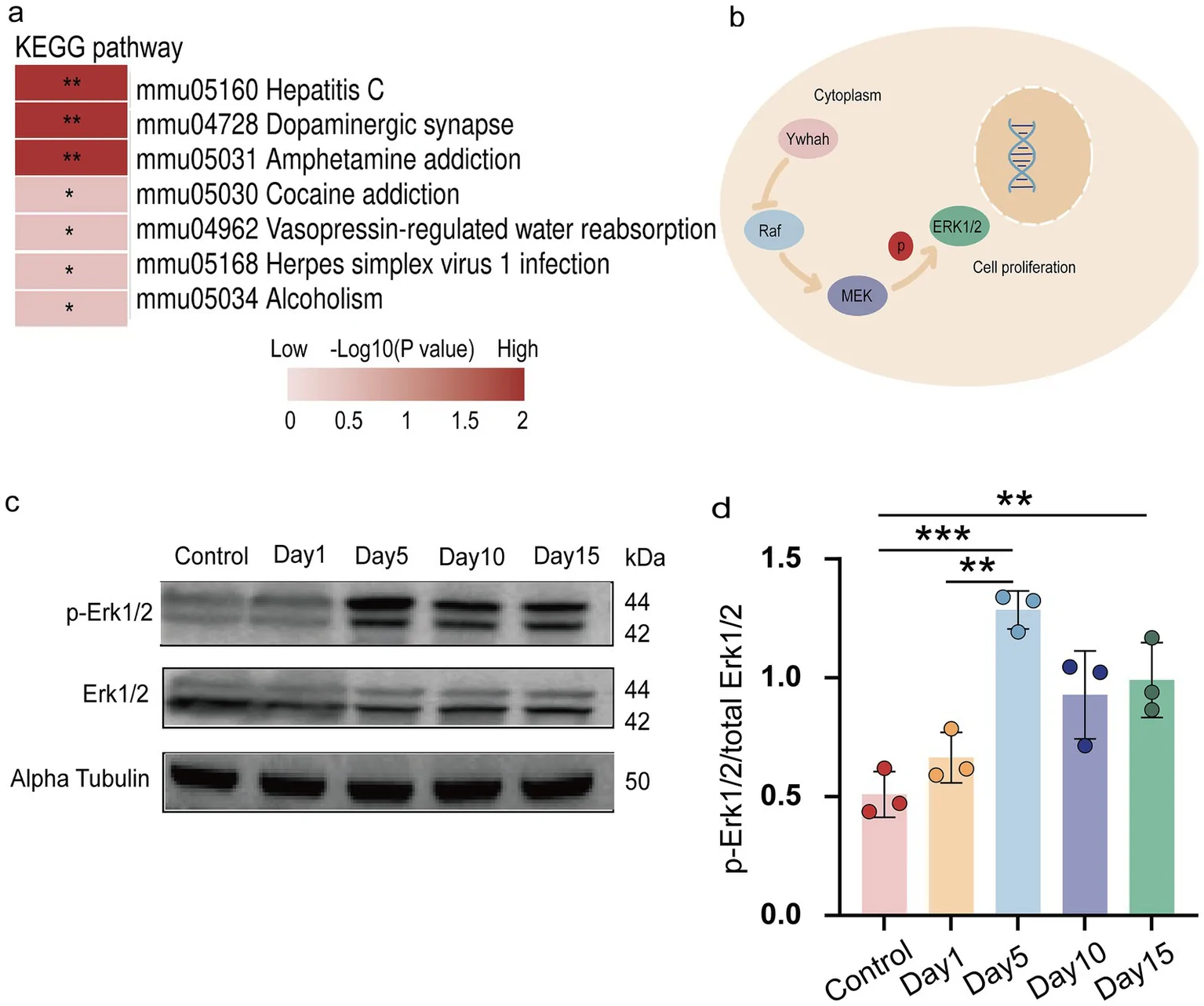
Effects of motion sickness habituation on signaling pathways in the vestibular region of the brainstem. **(a)** Functional clustering heatmap of proteins in the vestibular region of the brainstem, Control vs. Day 15 group; vertical axis indicates pathway annotations. **(b)** Simplified pathway diagram of Ywhah and Erk1/2 from proteomic KEGG enrichment analysis. **(c)** Western blot images. **(d)** Temporal changes in the ratio of phosphorylated Erk1/2 to total Erk1/2 during habituation. All data are presented as mean ± SD (*n* = 3). Statistical analyses were performed via one-way ANOVA with Tukey HSD *post hoc* test. *p < 0.05, **p < 0.01, ***p < 0.001.

## Discussion

In this study, we identified the vestibular region of the brainstem as the brain region with the most prominent molecular responses during motion sickness habituation through multi-brain-region proteomic comparison. Four behavioral indicators verified the successful establishment of the motion sickness habituation model. At the molecular level, Ywhah expression decreased progressively across the habituation time series, whereas the ratio of phosphorylated Erk1/2 to total Erk1/2 increased gradually. Both molecular alterations first reached statistical significance on Day 5, presenting a robust temporal correlation. This study integrated panoramic proteomics and molecular validation, demonstrating from multiple dimensions that the vestibular region of the brainstem may serve as a prioritized brain region for motion sickness habituation regulation, with Ywhah identified as a candidate protein for subsequent functional studies.

In the five-dimensional weighted scoring model, PCA separation, differential protein count, and unique protein count reflect the magnitude and specificity of proteomic changes, and were assigned 0.15 each. Protein interaction network and functional score reflect the functional organization and biological coherence of the altered proteins, and were assigned 0.275 each. The latter two were given higher weight because functional organization is more indicative of an active regulatory program than protein abundance alone. A brain region with many differentially expressed proteins that are functionally unrelated may not represent a coordinated regulatory response, whereas a region whose altered proteins converge on coherent functional categories and form a dense interaction network is more likely to represent an active regulatory program (Ideker et al., 2002). However, these five parameters are not fully independent. A brain region with more differentially expressed proteins would inherently contain more unique proteins, more opportunities for interactions, and more contributions to functional categories. Nevertheless, this interdependence affects all three brain regions equally. Although the total number of differentially expressed proteins differed by 7.6-fold between the hippocampus and the vestibular region of the brainstem, their unique protein proportions were nearly identical. If the hippocampus or cerebral cortex exhibited the most prominent proteomic response among the three regions examined, they should also show prominent scores across different dimensions, yet they did not. Therefore, this weighting scheme was not designed to validate five independent dimensions, but to provide a composite evaluation integrating complementary information. Its core value lies in capturing whether a brain region exhibits coordinated responses across multiple biological dimensions simultaneously. The vestibular region of the brainstem meets this characteristic.

Motion sickness habituation is an active neural remodeling process, rather than passive adaptation. The sensory conflict theory proposes that repeated rotational stimulation progressively updates the brain’s internal models encoding self-motion expectations, rematching expected signals with actual sensory afferents and thereby alleviating motion sickness responses (Reason and Brand, 1975). It should be noted that motion sickness habituation and vestibular compensation are two fundamentally distinct forms of neuroplasticity. Vestibular compensation refers to central functional reorganization following unilateral peripheral vestibular damage (El Mahmoudi et al., 2022), whereas motion sickness habituation refers to the progressive attenuation of motion sickness responses after repeated identical motion stimulation under intact bilateral vestibular function.

The brainstem contains nuclei involved in autonomic regulation, including the nucleus tractus solitarius and parabrachial nucleus, which have close fiber connections with the vestibular nuclei and together constitute nodes for sensorimotor integration and autonomic response regulation (Cai et al., 2007). In rats subjected to daily rotation, Fos-positive neurons in the caudal vestibular nucleus increased during the initial 7 rotations and returned to baseline after the 10th rotation, with a strong linear correlation between Fos-positive neuron numbers and defecation behavior (Cai et al., 2010). NMDA receptor signaling and GABAA receptor subunit expression in the vestibular nucleus also undergo temporal changes during habituation (Wang et al., 2012). These findings indicate that the brainstem vestibular region undergoes active molecular remodeling during motion sickness habituation.

Ywhah protein levels in the restraint-only control group exhibited no significant changes across the 15-day period, whereas the rotation group showed progressive downregulation. This confirms that the Ywhah downregulation observed in the rotation group is not attributable to repeated restraint or generalized stress adaptation, but is specifically associated with repeated vestibular stimulation. Previous studies have confirmed that repeated restraint stress alone can induce transcriptomic and HPA axis habituation, but its molecular signature differs from habituation-specific changes in the vestibular system (Waag et al., 2025). The experiment excluded restraint stress as a confounding factor, establishing Ywhah as a reliable candidate molecule during motion sickness habituation, and providing a necessary prerequisite for the subsequent validation of the temporal association between Ywhah and ERK.

Ywhah protein expression in the brainstem vestibular region showed progressive downregulation across the habituation time series, whereas the ratio of p-Erk1/2 to total Erk1/2 showed progressive upregulation, presenting an inverse trend with temporal coupling. This inverse trend suggests a possible functional association between Ywhah downregulation and elevated ERK phosphorylation. Existing literature has shown that 14–3-3 proteins can bind to RAF kinase and maintain it in a closed inactive conformation, thereby suppressing the MAPK/ERK signaling pathway (Liau et al., 2020). The ERK pathway is a cascade linking extracellular stimulation to intracellular transcriptional remodeling, governing neuronal plasticity and adaptive changes. Phosphorylation of ERK1/2 activates multiple downstream transcription factors that regulate the expression of synaptic plasticity-related genes, serving as an important molecular switch for neurons responding to environmental changes (Alonso et al., 2004; Selcher et al., 2003; Wiegert and Bading, 2011). Therefore, we propose a hypothesis that Ywhah downregulation may relieve RAF inhibition, allowing RAF to be recruited by RAS-GTP and form active dimers, which in turn activate MEK and ERK. This could explain the inverse trend between Ywhah downregulation and ERK phosphorylation elevation observed in this study.

Behavioral indicators fluctuated in the later phase of the 15-day training period, whereas Ywhah downregulation and ERK phosphorylation elevation remained stable after first reaching significance on Day 5, without parallel fluctuations corresponding to behavioral changes. This dissociation suggests that Ywhah and ERK signaling may primarily serve as the initiation program for habituation, rather than the sole mechanism for long-term maintenance. Synaptic plasticity studies indicate that early plasticity depends on post-translational modifications via kinase signaling, whereas late-phase maintenance requires new gene expression and structural remodeling (Katche et al., 2010). Accordingly, Ywhah and ERK changes may primarily serve as the initiation program for habituation, whereas long-term maintenance may require the recruitment of other compensatory circuits or structural neural plasticity.

This speculation is consistent with recent findings in the vestibular habituation field. Vestibular habituation training can induce recovery of dendritic length and spine density in the medial prefrontal cortex and increase dendritic complexity, suggesting that higher-order cortical structural plasticity participates in habituation maintenance (Fang et al., 2026). Temporal changes in NMDA receptor signaling and GABAA receptor subunit expression within the vestibular nucleus have also been documented during habituation, and these changes may constitute the molecular basis for maintaining the habituated state (Wang et al., 2012). Behavioral indicators reflect the animal’s overall behavioral output to rotational stimulation, regulated by the integrated activity of the HPA axis, autonomic nervous system, and motor systems, whereas Ywhah and ERK protein changes reflect the molecular program within the brainstem vestibular region. Thus, late-phase behavioral fluctuations may reflect a transitional period from molecular initiation to structural maintenance, during which maintenance mechanisms have not yet fully consolidated.

This framework also provides a logical basis for the disappearance of the habituated state. If Ywhah and ERK serve as the initiation signal for habituation, and long-term maintenance depends on structural plasticity, then as maintenance mechanisms decay over time, the habituated state will gradually dissipate.

Several limitations should be acknowledged for the present study, which need to be addressed in future investigations. Brain tissue blocks covering bilateral vestibular nuclei were dissected from the region between Bregma −5.40 mm and −7.10 mm according to the mouse brain stereotaxic atlas, which failed to achieve single-nucleus resolution. For this reason, the molecular changes described in this study reflect regional alterations within the vestibular region of the brainstem, rather than events restricted to individual vestibular subnuclei. The downregulation of Ywhah and the elevated ratio of phosphorylated Erk1/2 to total Erk1/2 only represent temporally correlated molecular events occurring during motion sickness habituation within the vestibular region of the brainstem, without confirmed causal relationships. Although relevant speculations are proposed based on published literature, these assumptions still require validation through causal functional experiments.

In summary, four behavioral indicators confirmed the successful establishment of the habituation model, which provided solid behavioral evidence for subsequent temporal analysis of molecular events. Proteomic comparison across multiple brain regions identified the vestibular region of the brainstem as the area exhibiting the most prominent molecular responses during motion sickness habituation. Subsequent temporal expression profiling of candidate molecules in this region characterized the dynamic patterns of Ywhah and the ratio of phosphorylated Erk1/2 to total Erk1/2 throughout the habituation process. Although further functional experiments are required to verify the causal relationships among these molecules, this study constructs a complete research framework covering behavioral temporal observation, multi-brain-region screening and molecular expression validation. The present work offers temporal reference data for exploring molecular mechanisms underlying motion sickness habituation and provides candidate proteins for therapeutic research on vestibular-related disorders.

## Data Availability

The raw data supporting the conclusions of this article will be made available by the authors, without undue reservation.

## Footnotes

1
http://eggnog5.embl.de/#/app/home

2
http://www.genome.jp/kegg/kaas/

3
http://www.kegg.jp/kegg/mapper.html

4
https://cn.string-db.org/
