# Correction: Time-course profiling of behavioral phenotypes and Ywhah/ERK signaling expression within the vestibular region of the brainstem during motion sickness habituation

**DOI:** 10.3389/fnins.2026.2026774

**Published:** 2026-10-29

**Authors:** Xue Yue, Chenxi Jia, Lihong Zhai, Jie Deng, Dongjun Yang, Zhanguo Jin

**Affiliations:** 1 Anhui Medical University, Hefei, Anhui, China; 2 Air Force Medical Center, PLA, Beijing, China

**Keywords:** brainstem, motion sickness habituation, neurophysiology, neuroplasticity, proteomics

An incorrect Funding statement was provided. The correct funding statement reads:

**Funding**

The author(s) declared that financial support was not received for this work and/or its publication.The original version of this article has been updated.

The original version of this article has been updated.

